# Short‐Term Outcomes of Two Interventions for Patients With Active Proliferative Diabetic Retinopathy in an Urban, Safety‐Net Hospital

**DOI:** 10.1002/hcs2.70109

**Published:** 2026-10-08

**Authors:** Paul S. Micevych, Abu Tahir Taha, Anika Kumar, Jay M. Stewart

**Affiliations:** ^1^ Department of Ophthalmology University of California, San Francisco San Francisco United States; ^2^ Department of Ophthalmology Zuckerberg San Francisco General Hospital and Trauma Center San Francisco United States; ^3^ Department of Ophthalmology & Visual Sciences University of Utah Salt Lake City United States; ^4^ Department of Ophthalmology & Visual Sciences University of California, Davis Sacramento United States

**Keywords:** pan‐retinal photocoagulation, process measure, proliferative diabetic retinopathy

## Abstract

**Background:**

Proliferative diabetic retinopathy carries significant visual morbidity and loss to follow‐up can lead to poorer visual outcomes. This study aims to evaluate the short‐term impact of two systems‐focused clinical initiatives directed toward reducing loss‐to‐follow‐up and increasing pan‐retinal photocoagulation (PRP) rates in patients with active proliferative diabetic retinopathy (PDR) in an urban, safety‐net, teaching‐hospital setting. The introduction of an additional weekly PRP clinic was followed by the creation of an active PDR patient registry with an appointment reminder system.

**Methods:**

This was a single‐center, observational, retrospective cohort study between December 2022 and May 2024. We established three cohorts of encounters based on the number of eyes with active PDR in sequential order: historical controls (115 encounters) in the 8 months preceding the first intervention, increased provider PRP volume (74 encounters) over the next 4 months, and patient registry with an appointment reminder system in addition to increased provider PRP volume (65 encounters) for the following 4 months. We used linear and generalized linear mixed‐effects models clustered at the patient level. Short‐term process measures included the number of monthly PRP clinics, time to PRP after referral, rates of PRP and full PRP, PRP clinic adherence rate, 3‐month ophthalmology clinic adherence rate, and 3‐month loss‐to‐follow‐up rate.

**Results:**

The introduction of an additional weekly PRP clinic was associated with a decrease in time from referral to PRP by 37 days (*p* < 0.001), increased odds of 3‐month PRP (odds ratio [OR]: 6.53; 95% confidence interval [CI]: 3.03–14.10; *p* < 0.001) and 3‐month full PRP (OR: 4.66; 95% CI: 2.22–9.78; *p* < 0.001). Subsequent introduction of a patient registry/appointment reminder system was associated with decreased odds of 3‐month loss‐to‐follow‐up (OR: 0.10; 95% CI: 0.01–0.79; *p* < 0.05) compared to historical cohort and improved odds of 3‐month ophthalmology clinic adherence (OR: 2.96; 95% CI: 1.18–7.47; *p* < 0.05) compared to the increased PRP clinic cohort.

**Conclusions:**

Dedicating additional clinic time for PRP and introducing a high‐risk registry and reminder system were associated with improved access to care, reduced loss‐to‐follow‐up, and increased overall clinic adherence in an urban, safety‐net hospital system. Long‐term visual and functional outcomes of these initiatives are an essential area of future study.

## Graphical abstract

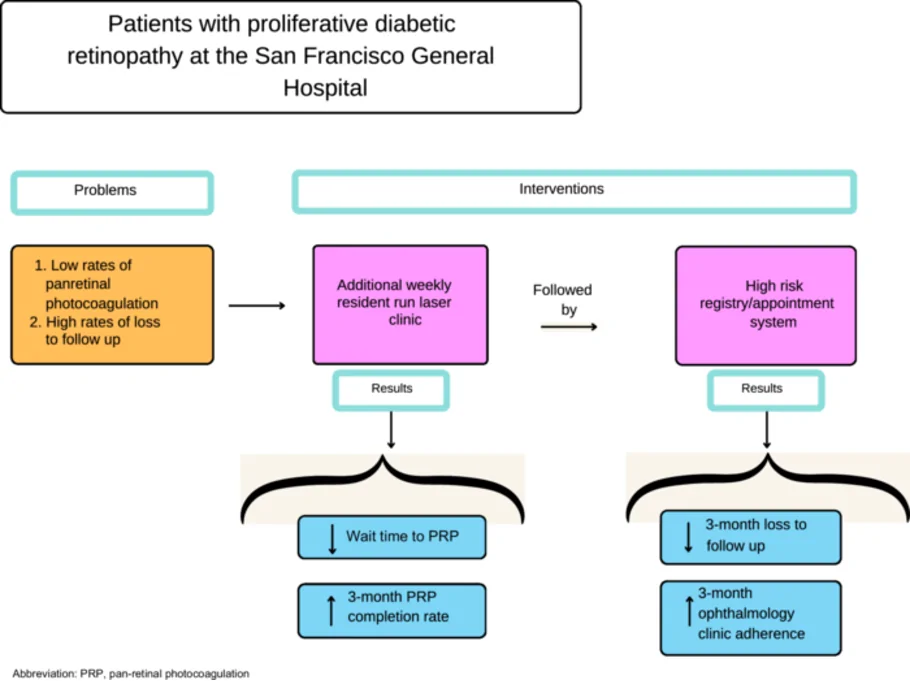

An additional weekly resident‐run panretinal photocoagulation (PRP) clinic and implementation of a high‐risk registry and appointment system were associated with a decreased wait time from proliferative diabetic retinopathy (PDR) diagnosis to PRP, increased 3‐month PRP completion rates, decreased 3‐month loss to follow‐up, and increased ophthalmology clinic adherence rate.

**Abbreviations**
anti‐VEGF: anti‐vascular endothelial growth factor
BCVA: best corrected visual acuity
CI: confidence interval
DR: diabetic retinopathy
logMAR: logarithm of the minimum angle of resolution
NH: non‐Hispanic
OR: odds ratio
PRP: pan‐retinal photocoagulation
PPV: pars plana vitrectomy
PDR: proliferative diabetic retinopathy
SD: standard deviation
ZSFG: Zuckerberg San Francisco General Hospital

## 1 Background

Complications related to diabetic retinopathy (DR) are increasingly prevalent causes of blindness and visual impairment worldwide [1]. Systematic reviews and meta‐analyses have shown that diabetic patients may be at higher risk of developing dry eye disease and may suffer from poorer visual and structural outcomes post‐cataract surgery [2, 3]. In the United States, urban and safety‐net hospital settings often manage a high burden of advanced diabetic disease. DR is a significant cause of vision impairment at Zuckerberg San Francisco General Hospital (ZSFG), the safety‐net hospital for the city and county of San Francisco, where the prevalence of DR is approximately 16% [4]. Neovascular complications of proliferative DR (PDR), the most advanced stage of DR, are a major cause of irreversible vision loss. Current effective medical treatment of proliferative disease involves anti‐vascular endothelial growth factor (anti‐VEGF) injections and pan‐retinal photocoagulation (PRP), with aggregated evidence suggesting better outcomes with anti‐VEGF therapy alone [5, 6]. However, the treatment modality may differ based on the sociodemographic factors of the patients being served. Nonetheless, despite the introduction of highly effective in‐office treatment, many patients develop neovascular complications requiring surgical management with pars plana vitrectomy (PPV). The most common complications of PDR that require PPV are non‐clearing or recurrent vitreous hemorrhage and tractional retinal detachment [7]. Prior work has demonstrated a 6% annual incidence of PPV among patients with PDR [8]. At ZSFG, our group previously demonstrated a host of individual and systems‐focused risk factors underlying progression to neovascular complications requiring PPV [9]. We found that the absence of PRP conferred nearly five times increased odds of PPV, and each month of loss‐to‐follow‐up during intervals of active PDR increased the odds of PPV by 10% [9]. Loss‐to‐follow‐up is a major problem in the care of patients with DR and is related to numerous social determinants of health [10, 11, 12]. Moreover, delays in care to PRP have been associated with poor visual outcomes in PDR [13]. Due to these concerns, anti‐VEGF monotherapy is typically not currently employed as a first‐line strategy in the management of PDR at ZSFG; instead, prompt 360‐degree PRP is the preferred management pattern for new PDR in this health system.

Most existing successful screening programs and initiatives focus on early detection and treatment of disease prior to proliferative disease [14]. Emerging evidence suggests systemic fibrate therapy may mitigate the incidence and progression of DR [15]. Fewer studies, however, have demonstrated evidence‐based successful strategies to optimize the systematic delivery of care among groups of patients with active PDR. Within the diverse, urban, safety‐net hospital patient population, it is unclear which types of system‐based interventions may have the greatest impact in improving patient outcomes with proliferative disease. Previously at ZSFG, a patient registry with a phone call reminder system was created for patients receiving intraoperative silicone oil tamponade to reduce silicone‐oil‐related complications and ensure timely removal when clinically appropriate [16]. The introduction of the system improved attendance at follow‐up visits and reduced the duration of silicone oil in patients' eyes [16].

Based on our prior work and the existing literature, the Department of Ophthalmology at ZSFG initiated a series of staged changes in 2023 to the clinical management of patients with active PDR to ameliorate structural and process measures. The staged, dual‐armed initiative specifically targeted weaknesses in care identified in the prior study related to PRP and loss‐to‐follow‐up [9]. The first arm of the initiative involved the opening of an additional weekly clinic dedicated to PRP, and the second involved the creation of a high‐risk patient registry and appointment reminder system for patients with active PDR. We seek to evaluate short‐term process measures of these staged interventions in this retrospective natural history study. We hypothesize that both interventions would be associated with positive impacts on short‐term systems‐focused process measures.

## 2 Methods

This work was a retrospective, single‐center, natural history study performed at ZSFG in San Francisco, California. Search criteria included all patients referred for PRP with a diagnosis of type 1 or 2 diabetes mellitus with active PDR, defined as PDR with signs of active neovascularization requiring treatment. We identified subjects for inclusion in the study upon referral to the PRP clinic, as a proxy for patients with active PDR. We included repeat referrals to the PRP clinic for future fill‐in sessions or fellow‐eye treatment. Primary exclusion criteria included any indication for photocoagulation other than active PDR. This research was approved by the Institutional Review Board of the University of California, San Francisco (approval number: 16‐20881), was performed in accordance with the Health Insurance Portability and Accountability Act regulations, and followed the tenets of the Declaration of Helsinki and its subsequent revisions. The requirement for informed consent was waived by the Institutional Review Board of UCSF, due to the retrospective nature of the study and lack of contact with, or risk to, the study subjects.

Our study comprised two serial changes implemented 4 months apart. Prior to the intervention, there was one existing, half‐day, weekly PRP laser clinic with an average of 4–6 available patient slots, and patients did not receive any form of clinic appointment reminders. The first intervention, which began on August 23, 2023, involved the introduction of an additional weekly, resident‐performed, half‐day PRP laser clinic template with an average of 4–6 additional patient slots. Clinic staff were notified of this intervention prior to its implementation to optimize staffing assignments and ensure the availability of rooms and equipment. The second intervention, which started on January 3, 2024, involved implementing a high‐risk patient registry and appointment reminder system for patients with active PDR. The active PDR patient registry was managed through REDCap (Vanderbilt University, Nashville, United States), a free Health Insurance Portability and Accountability Act‐compliant database, comprising data from all patients with active PDR referred for PRP who were identified at the time of referral. The appointment reminder system involved a weekly review of the patient registry. Each week, trained staff sent reminder phone calls to patients with appointments in the upcoming week, rescheduled no‐show patients from the preceding week, and offered verbal patient education as requested. All tasks were performed by trained, existing ophthalmic technicians in the ZSFG ophthalmology clinic. Financial support to expand hours for existing staff was covered through a ZSFG institutional grant. Certified phone interpreters available through ZSFG were used to ensure language‐concordant communication.

The authors manually reviewed the electronic medical records from December 2022 to May 2024. Based on the time of referral for PRP, we subclassified patients into three active PDR groups. For the approximately 8‐month window preceding the intervention between December 1, 2022, and August 22, 2023, we categorized patients into the historical control cohort. For the approximately 4‐month window between August 23, 2023, and January 1, 2024, we categorized patients into the increased PRP cohort. January 2 was a holiday, therefore no clinics were scheduled. For the approximately 4‐month window between January 3, 2024, and May 3, 2024, we categorized patients in the increased PRP with registry and reminder system cohort. We included a larger referral window for the historical control cohort than that for each treatment group to minimize the impact of natural or random variation.

At the time of referral for PRP, we identified pre‐specified baseline variables and process measures for a follow‐up period of 3 months. Baseline variables included age, sex, race/ethnicity, language, visual acuity, hemoglobin A1c, and PRP status. Primary short‐term 3‐month process measures, each designated as a binary variable, were: rates of 3‐month PRP rate, 3‐month full PRP rate, PRP clinic adherence rate, 3‐month ophthalmology clinic adherence rate, and 3‐month loss‐to‐follow‐up rate.

Time to PRP after referral was defined as the number of days between the referral date and the date on which patients received PRP. Of the patients referred for PRP within each cohort, we manually identified patients who received PRP or full PRP within the 3‐month period following the referral date to establish 3‐month PRP and 3‐month full PRP rates, respectively. 3‐month PRP was defined by documentation of incomplete treatment, whether intentional or not. 3‐month full PRP was defined as a 360‐degree laser treatment without notation of incomplete areas or a plan for multiple sessions. This classification was used in our group's prior published work with this patient population [9]. The PRP clinic adherence rate was determined by identifying the patients within each cohort who presented to their scheduled PRP appointment created at the time of referral. The 3‐month ophthalmology clinic appointment adherence rate was determined by identifying patients within each cohort who attended all of their ophthalmology clinic appointments within 3 months of the referral date. PRP clinic adherence is a critical metric reflecting nonadherence to a single procedural appointment of known high importance in preventing vision‐threatening complications of PDR. 3‐month ophthalmology clinic adherence reflects a broader assessment of clinic adherence. Lastly, the loss‐to‐follow‐up rate was determined by identifying patients within each cohort who had no ophthalmology visit (either PRP or clinic) within 3 months after the PRP referral.

We performed statistical analysis using the open‐source and free computing software R (R Foundation for Statistical Computing, Vienna, Austria). We used the chi‐square and Fisher's exact test of independence for categorical variables and the Kruskal–Wallis test for continuous variables to find mean baseline differences between patient and systems‐based variables. We used the “lme4” R package to determine the association between mean values of process measures and cohort assignment of patients (historical, increased provider volume, increased provider volume and high‐risk registry/appointment reminder system) [17]. Specifically, we used each patient encounter as the unit of analysis. We utilized linear mixed effects regression with a random intercept for patient ID for the continuous variable of “time to PRP clinic.” The addition of a nested structure of random intercepts, entailing primary patient ID and secondary eye ID, which would account for any correlation between the eyes of the same patient, did not demonstrate a better fit compared to a random intercept of patient ID alone when the two models were compared using model comparison analysis of variance. Thus, the simpler random intercept structure was chosen for all outcomes. For all binary categorical variables, we used a mixed‐effects logistic regression with a binomial family distribution in addition to a random intercept for patient ID. Additionally, we used the package “blme” in R Studio to apply Firth‐like penalization to address potential sparse data or separation for rare events. The number of monthly clinics was compared across the three cohorts using a Wilcoxon pairwise comparison. Statistical significance was set at *p* < 0.05 for all analyses.

## 3 Results

This study included a total of 254 encounters based on the number of eyes from 138 patients, comprised of 115 encounters from 81 patients in the historical control cohort, 74 encounters from 29 patients in the increased PRP cohort, and 65 encounters from 28 patients in the increased PRP with registry and reminder system cohort. Classification of encounter patterns can be observed in Supplemental Table S1. Table 1 represents baseline demographic information for subjects in the control and treatment groups, recorded at the time of referral for PRP. Baseline age, sex, race/ethnicity, language, and hemoglobin A1c were assessed at the patient level, while best corrected visual acuity and PRP status were assessed at the eye level. There were no statistically significant differences in baseline variables across groups. Standardized mean differences for baseline patient characteristics can be referenced in Supplemental Table S2, which was unrevealing of large differences between cohorts.

**Table 1:** Baseline characteristics of study participants.

Characteristic	Historical (*n* = 81)	Increased provider volume (*n* = 29)	Increased provider volume + registry/appointment reminder system (*n* = 28)	*p* a
Age (years)	55 ± 9	53 ± 13	57 ± 13	0.298
Sex				0.061
Male	44 (54)	23 (79)	17 (61)	
Female	37 (46)	6 (21)	11 (39)	
Diabetes mellitus type				> 0.999
Type 1	4 (5)	1 (3)	1 (4)	
Type 2	77 (95)	28 (97)	27 (96)	
Preferred spoken language				0.075
English	29 (36)	13 (45)	10 (36)	
Spanish	47 (58)	14 (48)	11 (39)	
Other	5 (6)	2 (7)	7 (25)	
Ethnicity				0.215
Hispanic/Latino	39 (48)	17 (59)	13 (46)	
NH Asian	9 (11)	6 (21)	9 (32)	
NH Black	12 (15)	2 (7)	2 (7)	
NH White	10 (12)	1 (3)	3 (11)	
Other^b^	11 (14)	3 (10)	1 (4)	
Baseline A1c (%)	8.55 ± 2.38	7.93 ± 2.68	8.62 ± 2.16	0.480
Unknown	2	3	3	
Baseline logMAR BCVA^c^	0.46 ± 0.28	0.47 ± 0.30	0.40 ± 0.28	0.281
Unknown	0	0	1	
PRP status^c^				0.379
Naive	48 (42)	38 (51)	32 (49)	
Fill‐in	67 (58)	36 (49)	33 (51)	

*Note:* Data are presented as mean ± SD and frequency (percentage). Chi‐square test of independence is used for expected cell count > 5, whereas Fisher's exact test is used for expected cell count < 5.

Abbreviations: A1c, hemoglobin A1c; BCVA, best corrected visual acuity; logMAR, logarithm of the minimum angle of resolution; NH, non‐Hispanic; PRP, pan‐retinal photocoagulation; SD, standard deviation.

^a^ Kruskal–Wallis rank sum test, Pearson's chi‐square test, or Fisher's exact test.

^b^ Includes patients who self‐identified as “American Indian/Alaskan Native,” “Native Hawaiian Pacific Islander,” “other,” “multiple races,” or declined to answer.

^c^ Values are based on the number of eyes.

The impact of the two‐staged interventions on each pre‐specified 3‐month process measure is described in Table 2 and visualized in Figure 1. Using the historical group as a reference, the time from referral to the PRP clinic was reduced significantly in the increased provider volume cohort (*β*: −37 days; 95% confidence interval [CI]: −46 to −29; *p* < 0.001). Patients in the increased provider volume cohort were more likely to undergo PRP (odds ratio [OR]: 6.53; 95% CI: 3.03 to 14.10; *p* < 0.001) and full PRP (OR: 4.66; 95% CI: 2.22 to 9.78; *p* < 0.001) within 3 months after referral (Table 2). With historical group as a reference, patients in the increased provider volume and registry/appointment reminder system cohort were more likely to present to their initial PRP appointment (OR: 3.72; 95% CI: 1.27 to 10.90; *p* = 0.017), less likely to be loss‐to‐follow‐up (OR after firth‐like penalization: 0.10; 95% CI: 0.01 to 0.79; *p* = 0.029), and more likely to adhere to their ophthalmology clinic appointments (OR: 2.23; 95% CI: 0.99 to 5.06; *p* = 0.054) over the subsequent 3 months (Table 2).

**Table 2:** Results of mixed‐effects models.

Group	PRP clinic adherence rate		Time from referral to PRP clinic		3‐month PRP rate		3‐month full PRP rate		3‐month ophthalmology clinic adherence rate		3‐month loss‐to‐follow‐up^b^	
	OR (95% CI)^a^	*p*	Estimate, days (95% CI)^a^	*p*	OR (95% CI)^a^	*p*	OR (95% CI)^a^	*p*	OR (95% CI)^a^	*p*	OR (95% CI)^a^	*p*
Reference group: historical												
Group												
Historical	—	—	—	—	—	—	—	—	—	—	—	—
Increased provider volume	2.30 (0.90 to 5.89)	0.083	−37 (−46 to −29)	**< 0.001**	6.53 (3.03 to 14.10)	**< 0.001**	4.66 (2.22 to 9.78)	**< 0.001**	0.71 (0.33 to 1.52)	0.386	0.69 (0.16 to 2.92)	0.612
Increased provider volume + registry/appointment reminder system	3.72 (1.27 to 10.90)	**0.017**	−34 (−42 to −25)	**< 0.001**	4.27 (2.02 to 9.02)	**< 0.001**	4.01 (1.82 to 8.84)	**< 0.001**	2.23 (0.99 to 5.06)	0.054	0.10 (0.01 to 0.79)	**0.029**
Reference group: increased provider volume												
Group												
Increased provider volume	—	—	—	—	—	—	—	—	—	—	—	—
Increased provider volume + registry/appointment reminder system	1.60 (0.47 to 5.52)	0.455	4 (−4.6 to 12)	0.364	0.65 (0.28 to 1.54)	0.333	0.86 (0.40 to 1.84)	0.695	2.96 (1.18 to 7.47)	**0.021**	0.02 (0.00 to 0.78)	**0.036**

Abbreviations: CI, confidence interval; OR, odds ratio; PRP, pan‐retinal photocoagulation; —, reference.

^a^ Coefficients with the label “Estimate” utilize linear mixed‐effects models; those with “OR” label use generalized linear mixed‐effects models. All models were unadjusted for additional covariates, as baseline characteristics did not display large differences between groups. The *p*‐values bolded denote statistical significance.

^b^ OR (CI) and *p*‐values adjusted via firth‐like penalized logistic regression to address potential sparse data or separation. Raw 3‐month loss‐to‐follow‐up events *n* (%) for historical, increased provider volume, and registry/appointment reminder system are 32 (27.8%), 10 (13.5%), 6 (9.2%), respectively, based on total patient encounters in each group.

**Figure 1:**
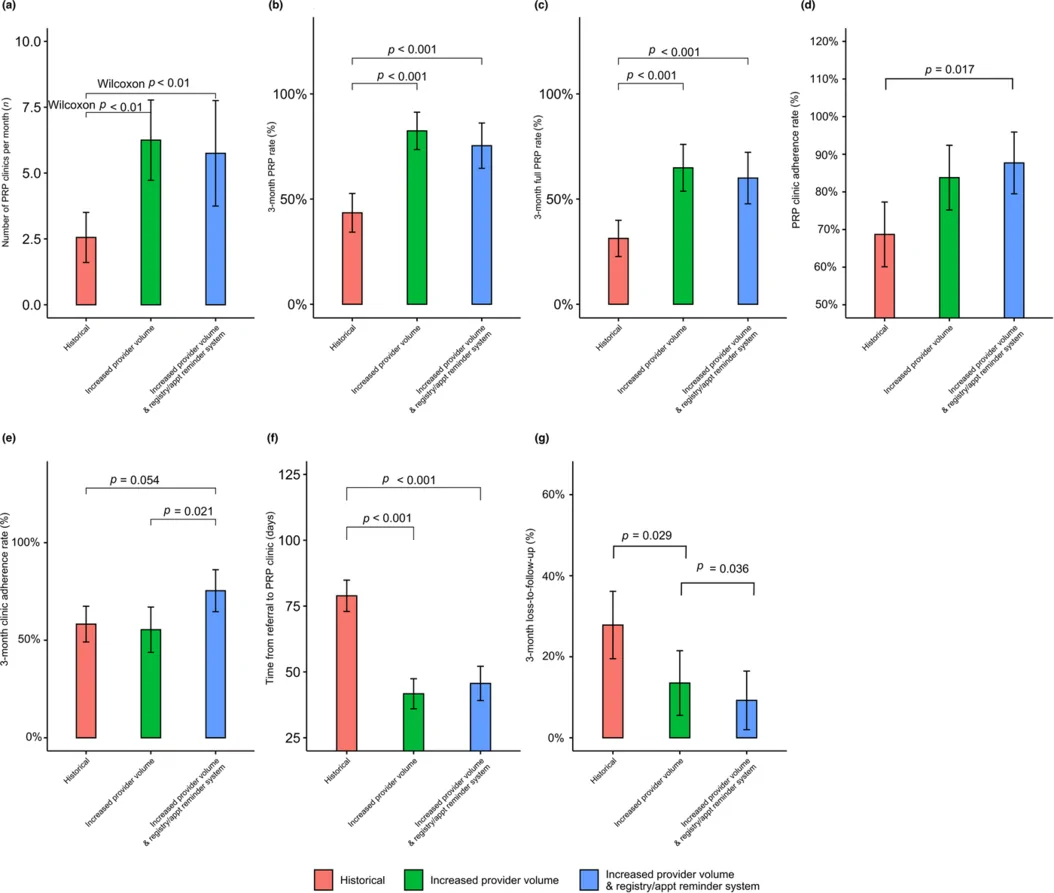
Error bar plots depicting comparisons in aggregate mean (95% CI) values and percentage rates of short‐term outcomes and processes between three cohorts. (a) The number of PRP clinics per month, (b) 3‐month PRP rate, and (c) 3‐month full PRP rate increased after the implementation of the increased provider volume initiative and after the registry/appointment reminder system intervention when compared to the historical group. The implementation of the registry/appointment reminder system was further followed by an improvement in (d) PRP clinic adherence rate and (e) 3‐month ophthalmology clinic adherence rate. Each intervention was associated with decreased (f) time from referral to PRP clinic and a reduction in (g) 3‐month loss‐to‐follow‐up. Except for figure (a), the displayed *p*‐values correspond to those of the mixed‐effects models in Table 2, which adjust for multiple visits by the same patient. A *p*‐value of < 0.05 denotes statistical significance. appt, appointment; CI, confidence interval; PRP, pan‐retinal photocoagulation.

Using the increased provider volume cohort as a reference, the additional implementation of a registry/appointment reminder system was associated with increased adherence to ophthalmology clinic appointments (OR: 2.96; 95% CI: 1.18 to 7.47; *p* = 0.021) and decreased odds of being loss‐to‐follow‐up (OR after firth‐like penalization: 0.02; 95% CI: 0.00 to 0.78; *p* = 0.036). Adjusted marginal effects corresponding to Table 2 can be found as Supplemental Table S3a,b and corresponding risk differences can be found in Supplemental Table S4a,b.

Figure 1 compares raw means of different outcomes between the three cohorts with corresponding *p*‐values obtained from the mixed‐effects models (Table 2), barring Figure 1a (Number of PRP clinics per month), which uses the Wilcoxon rank sum test. Notably, the mean (95% confidence limits) number of clinics per month increased from 2.6 (1.6 to 3.5) in the historical cohort to 6.3 (4.7 to 7.8) in the increased provider volume cohort (Wilcoxon *p* < 0.01; Figure 1a). A per month breakdown of clinics during the study period can be found in Supplemental Table S5. The mean (95% confidence limits) 3‐month PRP (Figure 1b) and 3‐month full PRP (Figure 1c) rates were 43.5% (34.3% to 52.7%) and 31.3% (22.7% to 39.9%), respectively, in the historical group, which increased to 82.4% (73.5% to 91.3%) and 64.9% (53.8% to 76.0%), respectively, in the increased provider volume cohort.

The mean (95% confidence limits) PRP clinic (Figure 1d) and 3‐month ophthalmology clinic appointment (Figure 1e) adherence rates were 68.7% (60.1% to 77.3%) and 58.3% (49.2% to 67.4%), respectively, in the historical group, which increased to 87.7% (79.5% to 95.9%) and 75.4% (64.6% to 86.2%), respectively, after the implementation of both increased volume and a registry/appointment reminder system; whereas the 3‐month loss‐to‐follow‐up rates (Figure 1g) decreased from 27.8% (19.5% to 36.1%) to 9.2% (2.0% to 16.4%).

The reduction in time from referral to PRP clinic visit is plotted in Figure 2. As visualized in the scatterplot, the time from referral declines and variation decreases over the staged interventions. No significant difference was found between the two intervention groups (*p* = 0.36).

**Figure 2:**
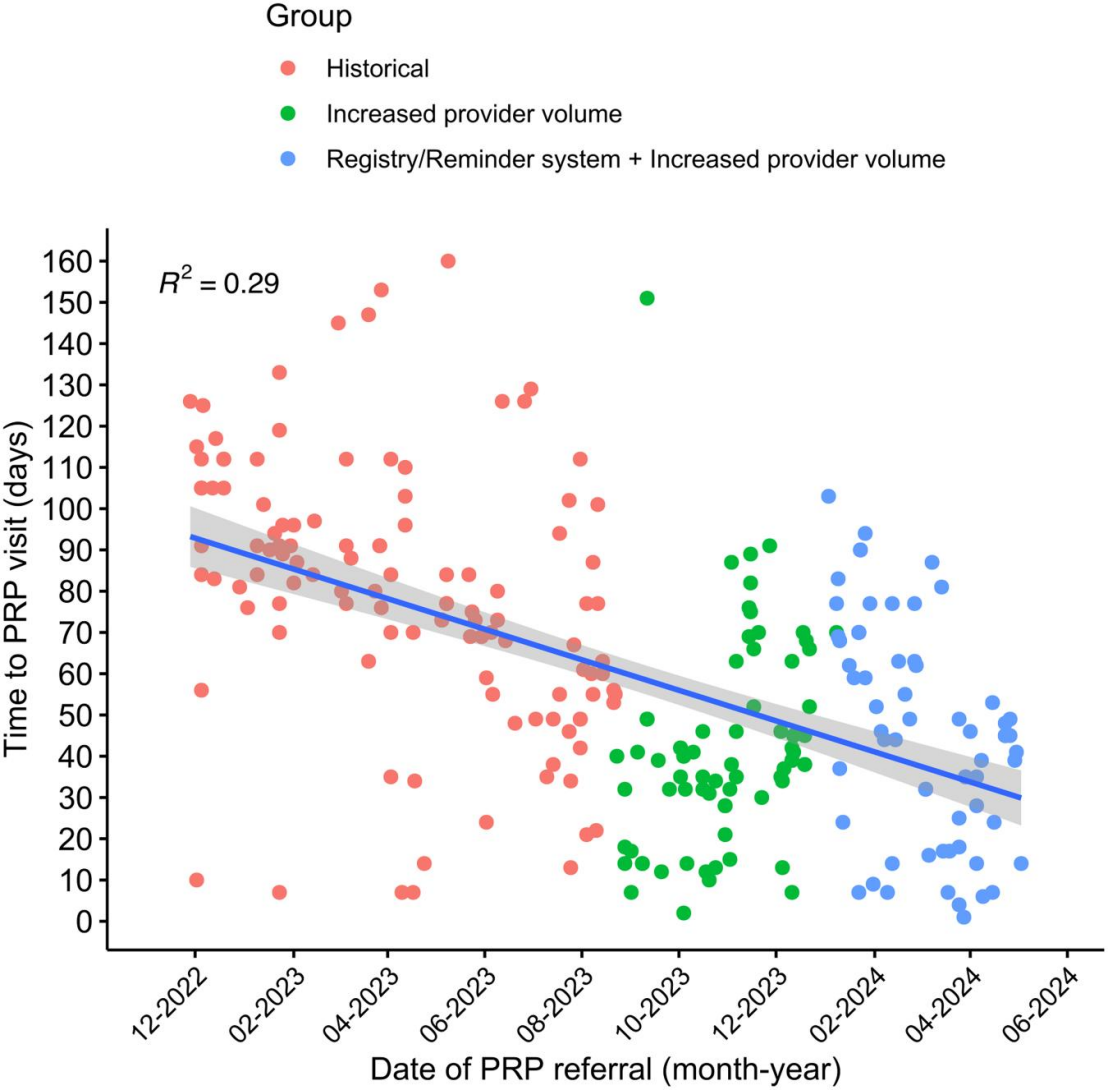
Time from referral date to PRP clinic plotted over the course of this study. Starting with the historical group in January 2023, the time to PRP visit decreased steadily during the two serial clinical interventions, and the amount of intra‐cohort variation decreased. The solid blue line represents a linear regression between the time from referral to PRP visit and the referral date. The shaded area around the blue line represents the 95% CI. Twenty‐nine percent of the variation in time to PRP visit can be explained by the months over which the study was conducted. CI, confidence interval; PRP, pan‐retinal photocoagulation.

## 4 Discussion

This study documents the impact of two serial clinical interventions instituted to improve process measures for patients with active PDR in an urban, safety‐net hospital. Collectively, our results show that increasing PRP clinic provider volume and implementing a registry/appointment reminder system were associated with improved short‐term process measures in patient care.

Our group previously found that the presence of full PRP decreased the odds of neovascular complications requiring diabetic vitrectomy fourfold in patients with active PDR [9]. Therefore, improving rates of full PRP and reducing delays to PRP were important aims of the first intervention. After establishing increased PRP volume, we observed a twofold increase in the mean 3‐month full PRP rates (31.3% vs. 64.9%; Figure 1e) and an approximate 50% reduction in mean time from the referral date to the PRP visit (79 vs. 42 days; Figure 1c).

The significant increase (43.5% vs. 82.4%) in 3‐month PRP rates following increased provider volume also merits discussion (Figure 1d). For reference, in the Diabetic Retinopathy Clinical Research Protocol S, 46% of the eyes required more than one sitting to achieve full PRP, the median time for which was 211 days (7 months) [18]. Our improved 3‐month PRP rate of 82% is notable, considering that limited evidence indicates full scatter PRP to be superior to mild scatter PRP regarding regression of neovascularization or improvement in visual acuity [19].

The results of the first intervention highlight the value and practicality of implementing prompt high‐volume PRP laser clinics in the management of PDR in the urban, safety‐net hospital system. Prior work has demonstrated that delays in PRP treatment may impact functional outcomes in DR, including visual acuity [13], and may be linked to lower socioeconomic status and inadequate health insurance coverage [20, 21]. Of mention, 94% of ZSFG patients are uninsured or recipients of Medicaid and Medicare programs [22]. As such, our results may be generalizable to high‐volume urban centers with similar diverse racial/ethnic and sociodemographic backgrounds. Increasing access to PRP may also reduce costs of care in limited‐resource hospital settings [23].

It is well known that active PDR patients, when left untreated, are at high risk of severe vision loss. The Early Treatment Diabetic Retinopathy Study report documented the incidence of severe vision loss in untreated “high‐risk” PDR patients to be 44% at 4 years [24]. Adherence to timely eye care and minimizing loss‐to‐follow‐up intervals carries paramount clinical significance for safety‐net hospital populations where loss‐to‐follow‐up rates may be higher than the average [10]. Our group previously showed that each additional month of loss‐to‐follow‐up during active PDR increased the risk of neovascular complications requiring diabetic vitrectomy by 10% [9].

Our second intervention, the patient registry/reminder system, was associated with reduced odds of loss‐to‐follow‐up (Figure 1g), increased odds of adherence to ophthalmology clinic appointments (although approaching statistical significance *p* = 0.054; Table 2), and improved the odds of PRP clinic adherence (Figure 1b). Whereas the expansion of PRP clinics in the first intervention likely reduced bottlenecks stemming from provider staffing shortages, introducing the patient registry/reminder system potentially mitigated sociodemographic hurdles relating to social determinants of health, education, miscommunication, and scheduling issues [25, 26]. Poor adherence to appointments is a multifactorial problem [25], and the factors involved can vary based on the geographical, racial/ethnic, and socioeconomic composition of the patient population [27, 28, 29, 30, 31, 32, 33]. Prior studies report that interventions such as reminder calls and assistance with scheduling may improve patient adherence to DR screening appointments [26]. Some studies have suggested that human reminders may be more effective than automated reminder systems [34, 35]. Additionally, the success rates of reminder systems may vary based on the sociodemographic characteristics of the patient population [34]. Our study suggests that this targeted intervention may improve adherence for high‐risk PDR patients for PRP clinics as well as general ophthalmology clinic appointments. Further investigation with larger cohorts and longer post‐intervention duration is needed to ascertain which type and style of reminder systems may be best suited for an urban, safety‐net hospital population. Notably, the operational burden of these interventions included increased pay for additional hours the clinic staff spent contacting patients and providing education. Moreover, expansion of ophthalmic technicians' hours to accommodate an additional clinic day per week is another financial cost to be wary of. These costs were absorbed by the hospital's institutional grant.

Our results should be interpreted with an understanding of their limitations. First, patient selection for active PDR patients was based on referral to PRP. This may have under‐identified the few PDR patients treated with anti‐VEGF monotherapy protocols. At ZSFG, such protocols are rare and typically reserved for patients with pathology delaying immediate referral for PRP, such as media opacity or severe diabetic macular edema. Providers at ZSFG typically refer for PRP promptly, even in the presence of such pathology, with the understanding that it may be resolved in the interim before the PRP visit. As such, we anticipate that the vast majority of patients with active PDR are represented in our cohorts. Furthermore, the relatively small‐to‐moderate sample sizes in intervention groups and the short follow‐up interval of 3 months reflect clinical volumes in our health system and the short duration between interventions, respectively. The larger sample size and duration in the historical group were intentional to establish a baseline for comparison and an attempt to account for a certain degree of natural fluctuation in provider and clinic availability. As illustrated in Figure 2, the time to PRP began to decline in advance of the initiation of any intervention. The secular trend observed in Figure 2 is likely a reflection of varying PRP clinic availability due to evolving resident schedules, holidays, and vacations. Moreover, unconscious behavioral modifications by residents and staff in anticipation of upcoming interventions may also explain this underlying trend. Such logistic factors in implementation science are difficult to eliminate fully; therefore, our results should be interpreted with caution in context of this significant limitation. Lastly, we believe long‐term evaluation of the functional impact of these interventions, such as complications of PDR (retinal detachments, neovascular glaucoma), change in best corrected visual acuity, and surgical interventions, is an essential area of future work; however, in the present study, the authors focused on short‐term processes.

## 5 Conclusions

Our two interventions were associated with improvement in various process measures that may optimize the health care delivery for active PDR patients in an urban, underserved population. We found that expanding PRP clinic volume and implementing a high‐risk registry with an appointment reminder system were associated with a reduction in time from referral to PRP visit, improvement in PRP and full PRP rates, increased adherence to ophthalmology appointments, and reduced loss‐to‐follow‐up. Future studies may focus on replicating this intervention with a larger sample size with longer pre‐ and post‐intervention periods in order to optimize care for patients with active PDR.

## Author Contributions

**Paul S. Micevych:** conceptualization, methodology, investigation, validation, supervision, data curation, writing – review and editing. **Abu Tahir Taha:** methodology, writing – review and editing, writing – original draft, data curation, formal analysis. **Anika Kumar:** data curation, investigation, project administration, writing – review and editing. **Jay M. Stewart:** funding acquisition, project administration, resources, supervision, writing – review and editing.

## Ethics Statement

This research was approved by the Institutional Review Board of the University of California, San Francisco (approval number: 16‐20881). The Institutional Review Board of the University of California, San Francisco is the Institutional Review Board of record for research conducted at Zuckerberg San Francisco General Hospital. This research was performed in accordance with the Health Insurance Portability and Accountability Act regulations, and followed the tenets of the Declaration of Helsinki and its subsequent revisions.

## Consent

The requirement for informed consent was waived by the Institutional Review Board of the University of California, San Francisco, due to the retrospective nature of the study and lack of contact with, or risk to, the study subjects.

## Conflicts of Interest

Jay Stewart serves as a consultant for Carl Zeiss, Merck, Valitor, Long Bridge, and Twenty Twenty, and holds a personal financial interest in Long Bridge. The remaining authors declare no conflicts of interest.

## Supporting information


Supporting File

## Data Availability

The data that support the findings of this study are available on request from the corresponding author. The data are not publicly available due to privacy or ethical restrictions.

## References

[1] J. L. Leasher , R. R. A. Bourne , S. R. Flaxman , et al., “Global Estimates on the Number of People Blind or Visually Impaired by Diabetic Retinopathy: A Meta‐Analysis From 1990 to 2010,” Diabetes Care 39, no. 9 (2016): 1643–1649, 10.2337/dc15-2171. 27555623

[2] K.‐Y. Chen , H.‐C. Chan , and C.‐M. Chan , “Is There a Link Between Dry Eye Disease and Diabetes Mellitus? A Systematic Review and Meta‐Analysis,” Journal of Diabetes and its Complications 39, no. 10 (2025): 109149, 10.1016/j.jdiacomp.2025.109149. 40829554

[3] K.‐Y. Chen , H.‐C. Chan , and C.‐M. Chan , “How Does Diabetes Shape the Landscape of Cataract Development and Surgical Success? A Systematic Review and Meta‐Analysis,” Endocrine 90, no. 2 (2025): 404–419, 10.1007/s12020-025-04374-w. 40775566

[4] A. Lim , J. Stewart , T.‐Y. Chui , et al., “Prevalence and Risk Factors of Diabetic Retinopathy in a Multi‐Racial Underserved Population,” Ophthalmic Epidemiology 15, no. 6 (2008): 402–409, 10.1080/09286580802435179. 19065433

[5] S. Sivaprasad , A. T. Prevost , J. C. Vasconcelos , et al., “Clinical Efficacy of Intravitreal Aflibercept Versus Pan‐Retinal Photocoagulation for Best Corrected Visual Acuity in Patients With Proliferative Diabetic Retinopathy at 52 Weeks (CLARITY): A Multicentre, Single‐Blinded, Randomised, Controlled, Phase 2b, Non‐Inferiority Trial,” Lancet 389 (2017): 2193–2203, 10.1016/S0140-6736(17)31193-5. 28494920

[6] K.‐Y. Chen , H.‐C. Chan , and C.‐M. Chan , “Which Treatment Modality Offers the Best Outcomes for Diabetic Retinopathy? A Systematic Review and Network Meta‐Analysis,” Diabetes Research and Clinical Practice 227 (2025): 112379, 10.1016/j.diabres.2025.112379. 40701389

[7] N. C. Patel , Y.‐T. Hsieh , C.‐M. Yang , et al., “Vitrectomy for Diabetic Retinopathy: A Review of Indications, Techniques, Outcomes, and Complications,” Taiwan Journal of Ophthalmology 14, no. 4 (2024): 519–530, 10.4103/tjo.tjo-d-23-00108. PMC11717329 39803397

[8] V. Schreur , J. Brouwers , R. A. C. Van Huet , et al., “Long‐Term Outcomes of Vitrectomy for Proliferative Diabetic Retinopathy,” Acta Ophthalmologica 99, no. 1 (2021): 83–89, 10.1111/aos.14482. PMC7891313 32643273

[9] P. S. Micevych , A. M. Taha , A. Poddar , and J. M. Stewart , “Individual and Systems‐Based Risk Factors for Diabetic Vitrectomy in an Urban Safety‐Net Hospital,” Ophthalmology Retina 7, no. 12 (2023): 1027–1034, 10.1016/j.oret.2023.05.014. 37236319

[10] M. Green , T. Tien , and S. Ness , “Predictors of Lost to Follow‐Up in Patients Being Treated for Proliferative Diabetic Retinopathy,” American Journal of Ophthalmology 216 (2020): 18–27, 10.1016/j.ajo.2020.03.023. 32243878

[11] R. Suresh , H. J. Yu , A. Thoveson , et al., “Loss to Follow‐Up Among Patients With Proliferative Diabetic Retinopathy in Clinical Practice,” American Journal of Ophthalmology 215 (2020): 66–71, 10.1016/j.ajo.2020.03.011. 32209344

[12] A. Obeid , X. Gao , F. S. Ali , et al., “Loss to Follow‐Up in Patients With Proliferative Diabetic Retinopathy After Pan‐Retinal Photocoagulation or Intravitreal Anti‐VEGF Injections,” Ophthalmology 125, no. 9 (2018): 1386–1392, 10.1016/j.ophtha.2018.02.034. 29606377

[13] M. Ohlhausen , C. Payne , T. Greenlee , A. X. Chen , T. Conti , and R. P. Singh , “Impact and Characterization of Delayed Pan‐Retinal Photocoagulation in Proliferative Diabetic Retinopathy,” American Journal of Ophthalmology 223 (2021): 267–274, 10.1016/j.ajo.2020.09.051. 33096065

[14] C. Fathy , S. Patel , P. Sternberg Jr. , and S. Kohanim , “Disparities in Adherence to Screening Guidelines for Diabetic Retinopathy in the United States: A Comprehensive Review and Guide for Future Directions,” Seminars in Ophthalmology 31 (2016): 364–377, 10.3109/08820538.2016.1154170. 27116205

[15] K.‐Y. Chen , H. C. Chan , and C.‐M. Chan , “Can Fibrate Therapy Redefine the Management of Diabetic Retinopathy? A Comprehensive Systematic Review and Meta‐Analysis of Efficacy and Safety,” Journal of Diabetes and Its Complications 39, no. 11 (2025): 109178, 10.1016/j.jdiacomp.2025.109178. 41038126

[16] D. Ma , W. Ma , X. Liu , and J. M. Stewart , “Improved Outcomes in Patients With Retinal Detachment After Implementation of a Silicone Oil Registry and Phone Call Reminder System,” Ophthalmology Retina 3, no. 7 (2019): 543–547, 10.1016/j.oret.2019.01.013. 31277794

[17] D. Bates , M. Mächler , B. Bolker , and S. Walker , “Fitting Linear Mixed‐Effects Models Using Lme4,” Journal of Statistical Software 67, no. 1 (2015): 1–48, 10.18637/jss.v067.i01.

[18] Writing Committee for the Diabetic Retinopathy Clinical Research Network , J. G. Gross , A. R. Glassman , et al., “Pan‐Retinal Photocoagulation vs Intravitreous Ranibizumab for Proliferative Diabetic Retinopathy: A Randomized Clinical Trial,” Journal of the American Medical Association 314, no. 20 (2015): 2137–2146, 10.1001/jama.2015.15217. PMC5567801 26565927

[19] T. Moutray , J. R. Evans , N. Lois , D. J. Armstrong , T. Peto , and A. Azuara‐Blanco , “Different Lasers and Techniques for Proliferative Diabetic Retinopathy,” Cochrane Database of Systematic Reviews 2018, no. 3 (2018): CD012314, 10.1002/14651858.CD012314.pub2. PMC6494342 29543992

[20] M. Lane , P. Mathewson , H. Sharma , et al., “Social Deprivation as a Risk Factor for Late Presentation of Proliferative Diabetic Retinopathy,” Clinical Ophthalmology 9 (2015): 347–352, 10.2147/OPTH.S73272. PMC4337620 25733801

[21] V. I. Lu , J. L. Patnaik , R. A. Scott , et al., “Risk Factors and Outcomes of Delayed Presentation of Diabetic Retinopathy Patients to a County Hospital,” Translational Vision Science & Technology 12, no. 9 (2023): 8, 10.1167/tvst.12.9.8. PMC10494984 37676677

[22] J. Wertheimer , “New Billing Policies at ZSFG (April 16, 2019),” Zuckerberg San Francisco General, accessed August 8, 2024, https://zuckerbergsanfranciscogeneral.org/news/new-billing-policies-zsfg-april-16-2019/.

[23] V. Rajeswaren , V. Lu , H. Chen , J. L. Patnaik , and N. Manoharan , “Healthcare Resource Utilization and Costs in an At‐Risk Population With Diabetic Retinopathy,” Translational Vision Science & Technology 13, no. 2 (2024): 12, 10.1167/tvst.13.2.12. PMC10876016 38359018

[24] Y. M. Paulus and M. S. Blumenkranz , “Proliferative and Nonproliferative Diabetic Retinopathy,” American Academy of Ophthalmology, accessed August 10, 2024, https://www.aao.org/education/current-insight/laser-treatment-of-proliferative-nonproliferative-.

[25] S. D. Solomon , R. Y. Shoge , A. M. Ervin , et al., “Improving Access to Eye Care,” Ophthalmology 129, no. 10 (2022): e114–e126, 10.1016/j.ophtha.2022.07.012. 36058739

[26] J. G. Lawrenson , E. Graham‐Rowe , F. Lorencatto , et al., “Interventions to Increase Attendance for Diabetic Retinopathy Screening,” Cochrane Database of Systematic Reviews 2018, no. 1 (2018): CD012054, 10.1002/14651858.cd012054.pub2. PMC6491139 29333660

[27] J. J. Peavey , S. L. D'Amico , B. Y. Kim , S. T. Higgins , D. S. Friedman , and C. J. Brady , “Impact of Socioeconomic Disadvantage and Diabetic Retinopathy Severity on Poor Ophthalmic Follow‐Up in a Rural Vermont and New York Population,” Clinical Ophthalmology 14 (2020): 2397–2403, 10.2147/OPTH.S258270. PMC7457718 32904606

[28] R. Yusuf , E. M. Chen , K. Nwanyanwu , and B. Richards , “Neighborhood Deprivation and Adherence to Initial Diabetic Retinopathy Screening,” Ophthalmology Retina 4, no. 5 (2020): 550–552, 10.1016/j.oret.2020.01.016. PMC8281953 32139294

[29] A. Olvera‐Barrios , M. Seltene , T. F. C. Heeren , et al., “Effect of Ethnicity and Other Sociodemographic Factors on Attendance at Diabetic Eye Screening: A 12‐Month Retrospective Cohort Study,” BMJ Open 11, no. 9 (2021): e046264, 10.1136/bmjopen-2020-046264. PMC8451288 34535475

[30] P. M. Hicks , A. R. Elam , M. A. Woodward , et al., “Perceptions of Respect From Clinicians by Patients in Racial and Ethnic Minority Groups With Eye Disease,” JAMA Ophthalmology 140, no. 2 (2022): 125–131, 10.1001/jamaophthalmol.2021.5371. PMC8678893 34913947

[31] E. C. Greig , R. Gonzalez‐Colaso , and K. Nwanyanwu , “Racial, Ethnic, and Socioeconomic Disparities Drive Appointment No‐Show in Patients With Chronic Eye Disease,” Journal of Racial and Ethnic Health Disparities 10, no. 4 (2023): 1790–1797, 10.1007/s40615-022-01363-x. PMC10392104 35864353

[32] E. Fairless and K. Nwanyanwu , “Barriers to and Facilitators of Diabetic Retinopathy Screening Utilization in a High‐Risk Population,” Journal of Racial and Ethnic Health Disparities 6, no. 6 (2019): 1244–1249, 10.1007/s40615-019-00627-3. PMC6880869 31463812

[33] J. M. Coney and A. W. Scott , “Racial Disparities in the Screening and Treatment of Diabetic Retinopathy,” Journal of the National Medical Association 114, no. 2 (2022): 171–181, 10.1016/j.jnma.2021.12.011. 35105457

[34] S. McLean , A. Booth , M. Gee , et al., “Appointment Reminder Systems Are Effective but Not Optimal: Results of a Systematic Review and Evidence Synthesis Employing Realist Principles,” Patient Preference and Adherence 10 (2016): 479–499, 10.2147/PPA.S93046. PMC4831598 27110102

[35] A. Parikh , K. Gupta , A. C. Wilson , K. Fields , N. M. Cosgrove , and J. B. Kostis , “The Effectiveness of Outpatient Appointment Reminder Systems in Reducing No‐Show Rates,” American Journal of Medicine 123, no. 6 (2010): 542–548, 10.1016/j.amjmed.2009.11.022. 20569761

