# Supplementary material for: Short‐Term Outcomes of Two Interventions for Patients With Active Proliferative Diabetic Retinopathy in an Urban, Safety‐Net Hospital
Source: Health Care Sci. 2026 Oct 8:10.1002/hcs2.70109. Online ahead of print. doi: 10.1002/hcs2.70109 (PMC13650190; doi:10.1002/hcs2.70109)
Supplement: Supplementary file 1 [file HCS2-9999-0-s001.docx]

Table S1. Patient encounter patterns.

| **Pattern** | **Patients, *n* (%)** | **Total observations** | **Observations per patient, mean (SD)** | **Range** |
| --- | --- | --- | --- | --- |
| Single observation | 69 (50%)^a^ | 69 | 1 (0.0) | 1–1 |
| Both eyes, single referral | 3 (2.2%)^b^ | 6 | 2 (0.0) | 2–2 |
| Same eye, multiple referrals | 32 (23.2%)^c^ | 74 | 2.3 (0.5) | 2–3 |
| Both eyes and multiple referrals | 34 (24.6%)^d^ | 105 | 3.1 (1.2) | 2–8 |

*Note*: Classification of repeat observations based on eye involvement and referral timing. Based on 138 unique patients.

^a^Single observation: patient appears only once in the dataset.

^b^Both eyes treated after a single referral date.

^c^Same eye referred on multiple different dates.

^d^Both eyes treated across multiple referral dates.

Abbreviation: SD, standard deviation.

Table S2. Standardized mean differences between groups.

| **Characteristic** | **Historical *vs.* increased provider volume** | **Historical *vs.* registry/reminder + increased provider volume** | **Increased provider volume *vs.* registry/reminder + increased provider volume^a^** |
| --- | --- | --- | --- |
| Age (years) | 0.20 | −0.16 | −0.31 |
| Baseline A1c (%) | 0.24 | −0.03 | −0.28 |
| Baseline logMAR BCVA | −0.05 | 0.21 | 0.26 |
| Diabetes mellitus type (%) | 0.07 | 0.07 | 0.01 |
| PRP status (%) | 0.19 | 0.15 | 0.04 |
| Preferred spoken language (%) | 0.14 | 0.31 | 0.29 |
| Race/ethnicity (%) | 0.24 | 0.25 | 0.22 |
| Sex (%) | 0.54 | 0.13 | 0.41 |

*Note*: For multicategorical variables Cohen’s *h* was calculated for each subcategory. Reported SMD are mean of absolute Cohen’s *h* values across all subcategories.

^a^SMD interpretation: < 0.1: trivial; 0.1–0.2: small; 0.2–0.5: moderate; 0.5–0.8: large; > 0.8: very large. Values > |0.2| may indicate meaningful imbalance based on underlying context.

Abbreviations: BCVA, best corrected visual acuity; logMAR, logarithm of the minimum angle of resolution; PRP, pan-retinal photocoagulation; SMD, standardized mean differences.

Table S3a. Adjusted marginal effects (predicted values).

| **Outcome** | **Scale**^a^ | **Historical** | **Increased provider volume** | **Registry/reminder + increased provider volume** |
| --- | --- | --- | --- | --- |
| PRP clinic adherence rate | Percentage (%) | 73.9 (59.6–88.2) | 85.3 (73.9–96.7) | 89.9 (79.7–100.1) |
| Time to PRP visit (days) | Days | 79 (73.7–84.3) | 41.7 (35.1–48.3) | 45.4 (38.4–52.4) |
| 3-month PRP rate | Percentage (%) | 43.7 (33.6–53.7) | 83.2 (74.0–92.5) | 76.5 (65.1–87.9) |
| 3-month full PRP rate | Percentage (%) | 29.8 (19.9–39.7) | 65.2 (52.9–77.5) | 61.8 (48.2–75.4) |
| 3-month clinic adherence rate | Percentage (%) | 61.3 (50.1–72.6) | 53.8 (40.3–67.3) | 76.9 (65.0–88.9) |
| 3-month loss-to-follow-up^b^ | Percentage (%) | 19.9 (7.8–32.1) | 17.9 (5.4–30.5) | 8.9 (−3.2–21.0) |

*Note*: Reference group: historical. Values shown as predicted (95% CI).

^a^Predicted values from mixed-effects models with random intercept for patient ID. Linear mixed model (time to PRP visit) shows predicted days. Generalized linear mixed models show predicted probabilities as percentages.

^b^Used Firth-like penalized logistic regression (bglmer with weakly informative priors) to address sparse data.

Abbreviations: CI, confidence interval; ID, identification; PRP, pan-retinal photocoagulation.

Table S3b. Adjusted marginal effects (predicted values).

| **Outcome** | **Scale**^a^ | **Increased provider volume** | **Registry/reminder + increased provider volume** |
| --- | --- | --- | --- |
| PRP clinic adherence rate | Percentage (%) | 87.2 (70.0–104.3) | 91.3 (75.9–106.7) |
| Time to PRP visit (days) | Days | 41.7 (35.9–47.5) | 45.6 (39.5–51.8) |
| 3-month PRP rate | Percentage (%) | 83.4 (72.6–94.1) | 76.6 (63.0–90.3) |
| 3-month full PRP rate | Percentage (%) | 65.1 (53.2–77.0) | 61.6 (48.1–75.1) |
| 3-month clinic adherence rate | Percentage (%) | 55.7 (42.2–69.3) | 78.0 (65.4–90.6) |
| 3-month loss-to-follow-up^b^ | Percentage (%) | 13.8 (6.6–20.9) | 6.2 (−0.4–12.8) |

*Note*: Reference group: increased provider volume. Values shown as predicted (95% CI).

^a^Predicted values from mixed-effects models with random intercept for patient ID. Linear mixed model (time to PRP visit) shows predicted days. Generalized linear mixed models show predicted probabilities as percentages.

^b^Used Firth-like penalized logistic regression (bglmer with weakly informative priors) to address sparse data.

Abbreviations: CI, confidence interval; ID, identification; PRP, pan-retinal photocoagulation.

Table S4a. Adjusted risk differences.

| **Outcome** | **Comparison** | **Risk** **difference** **(percentage** **points)^a^** |
| --- | --- | --- |
| PRP clinic adherence rate | Increased provider volume *vs.* Historical | 11.4% |
| PRP clinic adherence rate | Registry/reminder system + increased provider volume *vs.* historical | 16.0% |
| 3-month PRP rate | Increased provider volume *vs.* historical | 39.6% |
| 3-month PRP rate | Registry/reminder system + increased provider volume *vs.* historical | 32.8% |
| 3-month full PRP rate | Increased provider volume *vs.* historical | 35.4% |
| 3-month full PRP rate | Registry/reminder system + increased provider volume *vs.* historical | 32.0% |
| 3-month clinic adherence rate | Increased provider volume *vs.* historical | −7.6% |
| 3-month clinic adherence rate | Registry/reminder system + increased provider volume *vs.* historical | 15.6% |
| 3-month loss-to-follow-up^b^ | Increased provider volume *vs.* historical | −2.0% |
| 3-month loss-to-follow-up^b^ | Registry/reminder system + increased provider volume *vs.* historical | −11.0% |

*Note*: Reference group: historical.

^a^Risk difference = Predicted probability (comparison group) − predicted probability (reference group). Positive values indicate higher risk/probability in the comparison group.

^b^Loss-to-follow-up models used Firth-like penalized logistic regression (bglmer) to address sparse data.

Abbreviation: PRP, pan-retinal photocoagulation.

Table S4b. Adjusted risk differences.

| **Outcome** | **Comparison** | **Risk difference (percentage points)^a^** |
| --- | --- | --- |
| PRP clinic adherence rate | Registry/reminder system + increased provider volume vs increased provider volume | 4.1% |
| 3-month PRP rate | Registry/reminder system + increased provider volume vs increased provider volume | −6.7% |
| 3-month full PRP rate | Registry/reminder system + increased provider volume vs increased provider volume | −3.5% |
| 3-month clinic adherence rate | Registry/reminder system + increased provider volume vs increased provider volume | 22.3% |
| 3-month loss-to-follow-up^2^ | Registry/reminder system + increased provider volume vs increased provider volume | −7.6% |

*Note*: Reference group: increased provider volume.

^a^Risk difference = predicted probability (comparison group) − predicted probability (reference group). Positive values indicate higher risk/probability in the comparison group.

^b^Loss-to-follow-up models used Firth-like penalized logistic regression (bglmer) to address sparse data.

Abbreviation: PRP, pan-retinal photocoagulation.

Table S5. PRP clinics during study period.

| PRP clinics per month | Cohort assignment |
| --- | --- |
| 1 | 1 |
| 1 | 1 |
| 1 | 1 |
| 3 | 1 |
| 4 | 1 |
| 3 | 1 |
| 3 | 1 |
| 3 | 1 |
| 4 | 1 |
| 7 | 2 |
| 7 | 2 |
| 6 | 2 |
| 5 | 2 |
| 6 | 3 |
| 6 | 3 |
| 4 | 3 |
| 7 | 3 |

*Note*: 1: historical cohort; 2: increased PRP clinic volume; 3: registry and appointment reminder system + increased PRP clinic volume.

Abbreviation: PRP, pan-retinal photocoagulation.
